# Spatial variability in Arctic–boreal fire regimes influenced by environmental and human factors

**DOI:** 10.1038/s41561-024-01505-2

**Published:** 2024-09-02

**Authors:** Rebecca C. Scholten, Sander Veraverbeke, Yang Chen, James T. Randerson

**Affiliations:** 1 Faculty of Science, Vrije Universiteit Amsterdam, Amsterdam, the Netherlands; 2 Department of Earth System Science, University of California, Irvine, CA USA; 3 School of Environmental Sciences, University of East Anglia, Norwich, UK

**Keywords:** Natural hazards, Biogeochemistry, Environmental impact, Fire ecology, Boreal ecology

## Abstract

Wildfire activity in Arctic and boreal regions is rapidly increasing, with severe consequences for climate and human health. Regional long-term variations in fire frequency and intensity characterize fire regimes. The spatial variability in Arctic–boreal fire regimes and their environmental and anthropogenic drivers, however, remain poorly understood. Here we present a fire tracking system to map the sub-daily evolution of all circumpolar Arctic–boreal fires between 2012 and 2023 using 375 m Visible Infrared Imaging Radiometer Suite active fire detections and the resulting dataset of the ignition time, location, size, duration, spread and intensity of individual fires. We use this dataset to classify the Arctic–boreal biomes into seven distinct ‘pyroregions’ with unique climatic and geographic environments. We find that these pyroregions exhibit varying responses to environmental drivers, with boreal North America, eastern Siberia and northern tundra regions showing the highest sensitivity to climate and lightning density. In addition, anthropogenic factors play an important role in influencing fire number and size, interacting with other factors. Understanding the spatial variability of fire regimes and its interconnected drivers in the Arctic–boreal domain is important for improving future predictions of fire activity and identifying areas at risk for extreme events.

## Abstract (web summary)

Arctic–boreal biomes vary regionally in the sensitivity of their fire regime to climate, according to an analysis of properties of individual fires measured by satellite radiometry.

## Main

Many Arctic and boreal regions have experienced unprecedented fire activity in the past decade. Major regional fire complexes occurred in eastern Siberia during 2019, 2020 and 2021, in Alaska in 2015 and 2022, and in Canada in 2014 and 2023. Most recently, in the summer of 2023, a substantial expanse of Canada witnessed a record level of burning that reversed long-term trends in carbon storage within forests^1^. Extreme fire seasons in these regions were caused primarily by warm summer temperatures and high lightning activity^2,3^. Furthermore, land–atmosphere feedbacks due to changes in snowmelt timing, as well as shifts in the polar jet stream linked to a warming climate, have been shown to influence these large fire events^4,5^. There is ample evidence that recent boreal fire extremes are driven by climate change^2,4,6,7^. However, little is known as to why different regions repeatedly experience unprecedented extremes, while fire activity in other boreal areas remains relatively constant.

At a global scale, spatial variability in fire activity across biomes is driven by differences in vegetation, climate and human impact^8–11^. While global studies have revealed notable variability in Arctic–boreal regions, the underlying causes have not yet been explored. Various regional studies have highlighted the importance of extreme climatic conditions conducive to fuel drying and fire spread for extreme fire seasons in boreal regions^2,7,12^. Furthermore, ignition limitations govern Arctic–boreal fire occurrences^10,13^. However, most studies of contemporary and future Arctic–boreal fire activity have focused on the fire-prone regions of western North America and eastern Siberia, and less is known about what drives fire activity in other Arctic–boreal regions, including those with a strong anthropogenic influence. To date, we have missed a comprehensive understanding of the driving factors of spatial variability in Arctic–boreal fire activity. Understanding how climate, fuels and human activity spatially vary, interact and shape Arctic–boreal fire regimes is necessary to improve predictions of future Arctic–boreal fire activity.

Pyroregions are regions characterized by a similar fire regime^14^ and are defined by the long-term variability of fire number, size, intensity, duration, burned area, and timing and length of the fire season^8,15^. Fire-regime properties can substantially differ between ecoregions. It is thus crucial to assess the sensitivity of these different fire-regime properties to climatic and anthropogenic drivers, as well as fuel amount and composition. Due to the absence of accurate and long-term records of individual fires in many areas of the Arctic–boreal region, studies evaluating Arctic–boreal fire activity often rely on satellite-derived products of active fires and burned area. While these raster products are useful for assessing regional burned area and fire occurrence and intensity, they do not offer insights about the ignition location and timing, the sub-daily spread rate and the temporal evolution of individual fires. While some near-global datasets are available that derived such object-based fire information from remotely sensed burned areas^16–19^, they do not provide full spatial coverage of Arctic tundra and boreal forest biomes and have often not been optimized for fire dynamics in these regions. The Visible Infrared Imaging Radiometer Suite (VIIRS) launched in 2012 provides global active fire data at a 375 m spatial resolution and with a sub-daily revisit time. These data enable detailed tracking of individual fires and offer new insights into fire behaviour, as demonstrated, for example, by the Fire Events Database for California^20^.

In this Article, we develop an Arctic–boreal fire atlas^21^ using a fire event tracking system^22^ based on VIIRS active fire locations from the Suomi National Polar-orbiting Partnership satellite, which records fire growth in half-daily intervals. This fire atlas contains information about every recorded fire detected by at least one VIIRS observation between 2012 and 2023 within the circumpolar Arctic and boreal biomes. We recorded the number of ignitions and their location and timing, 12 hour spread rates, and the final fire size, duration and intensity for each fire. We aggregated or averaged fire characteristics from the individual fires into grid cells of 100 by 100 km to assess spatial variability in fire dynamics across the Arctic–boreal domain. Since science-quality archive data from VIIRS was available only between 2012 and 2021, we used only these years for this characterization of fire regimes. On the basis of the maps of fire-regime properties, we identified seven distinct pyroregions using an unsupervised clustering algorithm. We further evaluated the influence of anthropogenic and climatic drivers, including the role of fuel availability and lightning, on these pyroregions.

## Patterns of fire activity in Arctic–boreal regions

**Fig. 1:**
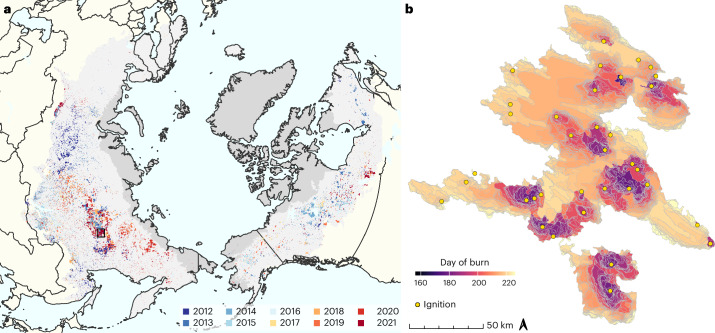
Arctic–boreal fire atlas. **a**, Fires in the Arctic–boreal biomes from 2012 to 2021. Fire perimeters are labelled by their year of burning. Arctic and boreal biome boundaries according to the World Wildlife Fund Terrestrial Ecoregions are shaded in dark and light grey. **b**, Fire spread for the largest fire in the database, which was recorded in Yakutia, eastern Siberia (127.4° E, 62.8° N), between 5 June and 15 September 2021. Yellow circles represent fire ignition locations. The black rectangle in **a** represents the location of the fire in **b**. Basemap in **a** from Natural Earth.

**Extended Data Fig. 1:**
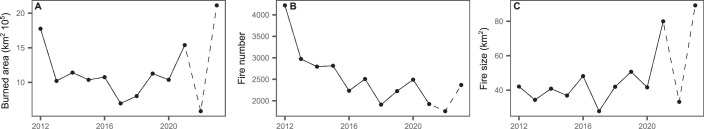
Time series of total burned area, the number of fires, and average fire sizes in Arctic and boreal biomes from 2012-2021. Fire number refers to the number of fire starts in each year. (**A**), total burned area. Fire numbers (**B**) show a decreasing trend (two-sided t-test of slope: −139 fires/year, p = 0.004), while average fire size (**C**) has increased by 2.9 km^2^ per year (two-sided t-test, p = 0.059). Dashed lines refer to data based on near real-time active fire locations instead of the science quality archive of active fire products.

From 2012 to 2021, we recorded 26,504 fires burning a total area of 1.12 million km^2^ in Arctic–boreal forest and tundra regions (Fig. 1a). Among these fires, we detected 11 that were larger than 5,000 km^2^. The largest fire documented in our database occurred in eastern Siberia in 2021, encompassed 35 separate ignition locations and burned an area of 15,759 km^2^ (Fig. 1b). Burned area varied considerably from year to year, from 70,000 km^2^ in 2017 to 180,000 km^2^ in 2012 (Extended Data Fig. 1). Near real-time VIIRS data revealed that 2023 set a new record, burning 210,000 km^2^ by the end of October. Notably, years with large burned areas did not always coincide with years with many fires. While fire numbers showed a significantly decreasing decadal trend (−138 fires yr^–1^, *P* = 0.004), fire sizes increased by 2.9 km^2^ per year (*P* = 0.059) between 2012 and 2023.

**Fig. 2:**
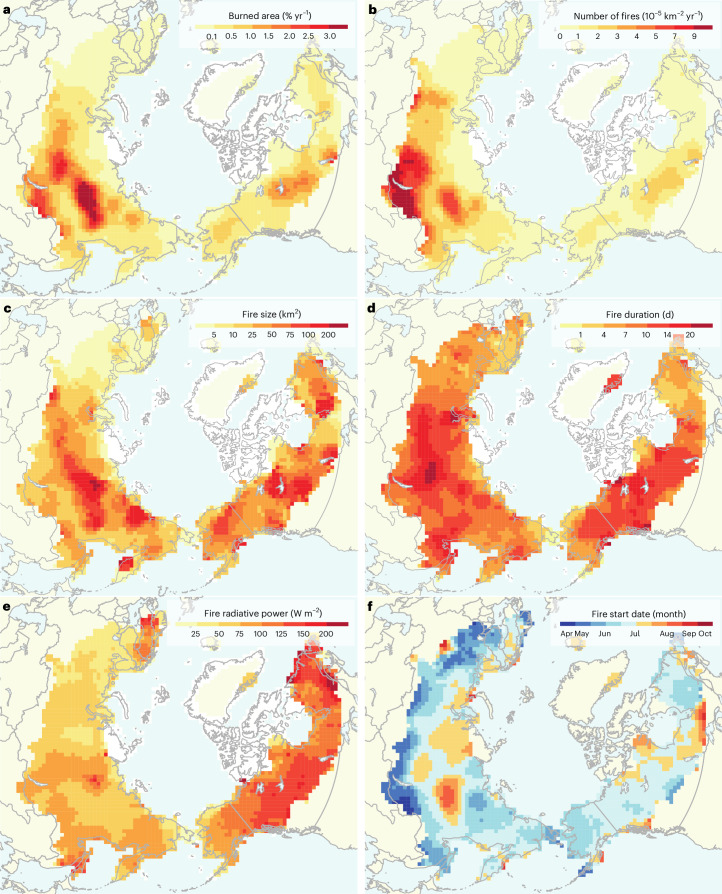
Circumpolar maps of fire characteristics derived from the Arctic–boreal fire atlas using satellite data from 2012 to 2021. **a**, Annual percentage burned area. **b**, Fire number density. **c**, Average fire size, **d**, Average fire duration. **e**, Average 95th percentile of fire radiative power per fire from VIIRS. **f**, Average start month of fires (April–October). Grid-cell dimensions are 100 by 100 km. Basemaps from Natural Earth.

Areas with high fire frequency were concentrated in several hotspot regions in the continental interior of Eurasia and North America, including in central, eastern and southern Siberia and western Canada (Fig. 2). Low annual burned areas occurred in western Eurasia and northeastern Canada and in northern tundra regions. Burned area was highest overall in regions where a high density of fire number co-occurred with large fire sizes (Fig. 2b,c). Eurasia displayed distinct latitudinal gradients in fire density and the timing of fire starts (Fig. 2d,f), with many early-season fires occurring in southern Siberia and the largest fires burning in northern forests. The regions with the highest fire number density were identified around Lake Baikal in southern Siberia and in Yakutia. By contrast, the largest fires occurred in central and northeastern Siberia, as well as in the Northwest Territories and Quebec in Canada. There were substantial differences in fire intensities between continents (Fig. 2e), with the highest fire radiative power recorded in Canada and the lowest in European Russia and western Siberia.

## Arctic–boreal pyroregions

**Fig. 3:**
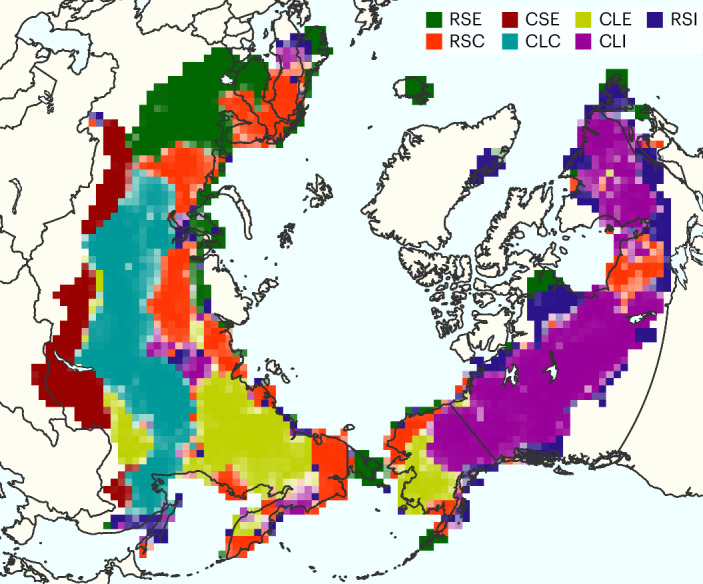
Geographical distribution of Arctic–boreal pyroregions. The cluster uncertainty is expressed through the transparency of each grid cell. RSE, rare-small-early; RSC, rare-small-cool; CSE, common-small-early; CLC, common-large-cool; CLE, common-large-early; CLI, common-large-intense; RSI, rare-small-intense. See Table 1 for the fire and environmental characteristics of the pyroregions. Basemap from Natural Earth.

**Extended Data Fig. 2:**
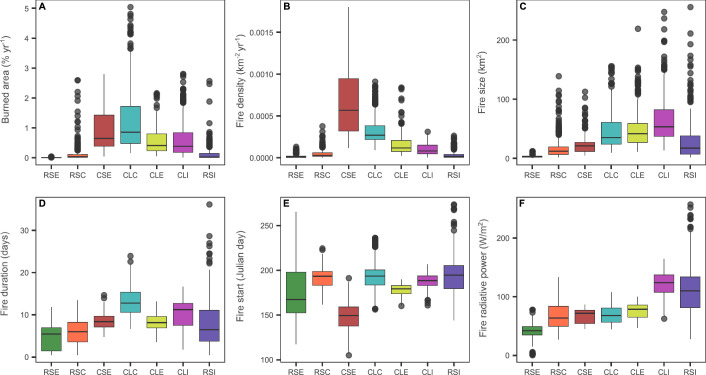
Fire characteristics of Arctic-boreal pyroregions. **a**, annual percentage burned area, **b**, fire number density, **c**, average fire size, **d**, average fire duration in pixel, **e**, average fire start day in pixel, **f**, average of the 95^th^ percentile of fire radiative power values per fire. Horizontal lines represent the median, and the lower and upper ends of each box are the 25th and 75th percentiles. Whiskers extend up to 1.5 times the interquartile range, sample points (grid cells) beyond that are represented as dots. RSE: rare-small-early (n = 328), RSC: rare-small-cool (n = 456), CSE: common-small-early (n = 185), CLC: common-large-cool (n = 394), CLE: common-large-early (n = 320), CLI: common-large-intense (n = 568), RSI: rare-small-intense (n = 297). See Fig. 3 for the geographical extent of the pyroregions.

Using the Arctic–boreal fire atlas and a clustering approach, we identified seven Arctic–boreal pyroregions with unique combinations of fire number density, fire size, fire intensity, area burned, and fire start timing and duration (Table 1 and Extended Data Fig. 2). The pyroregions were mostly geographically contiguous (Fig. 3). We named the pyroregions on the basis of fire frequency (rare/common), fire size (small/large) and intensity or fire start timing (cool/intense/early). Pyroregions with low fire number densities, namely rare–small–early (RSE), rare–small–cool (RSC) and rare–small–intense (RSI), also exhibited small fire sizes and were located predominantly in circumpolar northern tundra regions and northern Europe (Fig. 3). We identified three pyroregions characterized by common and large fires: common–large–cool (CLC) in western and central Siberia, common–large–early (CLE) in the eastern Siberian Republic of Sakha and western Alaska, and common–large–intense (CLI) across much of central Alaska and western and eastern Canada. CLI comprised the largest fires on average. Southern Siberia was notably distinct from other boreal pyroregions, displaying common, small and early-season fires (common–small–early, CSE).

**Table 1:** Average and standard deviation of fire and environmental characteristics of Arctic–boreal pyroregions

	Burned area	Total burned area	Fire number density	Fire size	Fire duration	Fire radiative power	Fire start	VPD	Lightning strike density	Above-ground biomass	Wilderness fraction
	(% yr^−1^)	(10^5^ km^2^ yr^-1^)	(*n* × 10^−5^ km^−2^ yr^−1^)	(km^2^)	(d)	(W m^−2^)	(Julian day)	(kPa)	(10^−5^ km^−2^ d^−1^)	(Mg ha^–1^)	(%)
**RSE**	0.01 ± 0.01	0.01 ± 0.01	0.17 ± 0.21	3.1 ± 1.9	4.8 ± 3.0	39.8 ± 15.6	174 ± 29	0.49 ± 0.19	5.41 ± 6.01	45.4 ± 41.3	28.8 ± 39.5
**RSC**	0.12 ± 0.29	0.41 ± 0.25	0.47 ± 0.47	16.5 ± 17.4	6.1 ± 2.9	68.4 ± 23.0	192 ± 10	0.48 ± 0.17	1.55 ± 2.07	21.7 ± 23.4	64.5 ± 36.9
**CSE**	0.93 ± 0.73	1.13 ± 0.89	6.85 ± 4.47	23.1 ± 17.6	8.5 ± 1.8	67.0 ± 12.1	148 ± 17	0.74 ± 0.16	7.49 ± 5.01	64.7 ± 27.7	20.3 ± 30.0
**CLC**	1.27 ± 1.04	4.50 ± 3.10	3.25 ± 1.61	45.8 ± 30.0	13.1 ± 3.4	69.1 ± 13.8	194 ± 16	0.74 ± 0.16	3.49 ± 3.05	54.3 ± 25.9	57.9 ± 33.8
**CLE**	0.56 ± 0.44	1.36 ± 1.16	1.59 ± 1.29	49.3 ± 31.6	8.2 ± 1.8	76.4 ± 12.4	178 ± 6	0.51 ± 0.14	0.86 ± 1.18	20.0 ± 80.0	80.1 ± 18.2
**CLI**	0.59 ± 0.58	3.42 ± 3.15	1.02 ± 0.72	63.0 ± 36.7	10.1 ± 3.6	122.0 ± 19.1	188 ± 7	0.58 ± 0.17	2.50 ± 3.46	34.9 ± 24.2	86.2 ± 20.4
**RSI**	0.13 ± 0.28	0.21 ± 0.31	0.35 ± 0.48	30.9 ± 38.0	7.9 ± 5.8	113.8 ± 44.4	196 ± 23	0.48 ± 0.20	2.23 ± 3.82	32.6 ± 35.2	64.9 ± 35.3

Fire-regime properties are based on data between 2012 and 2021. VPD and lightning strike density are multi-year (2012–2021) averages over the boreal fire season (March–October). RSE, rare-small-early; RSC, rare-small-cool; CSE, common-small-early; CLC, common-large-cool; CLE, common-large-early; CLI, common-large-intense; RSI, rare-small-intense.

**Extended Data Fig. 3:**
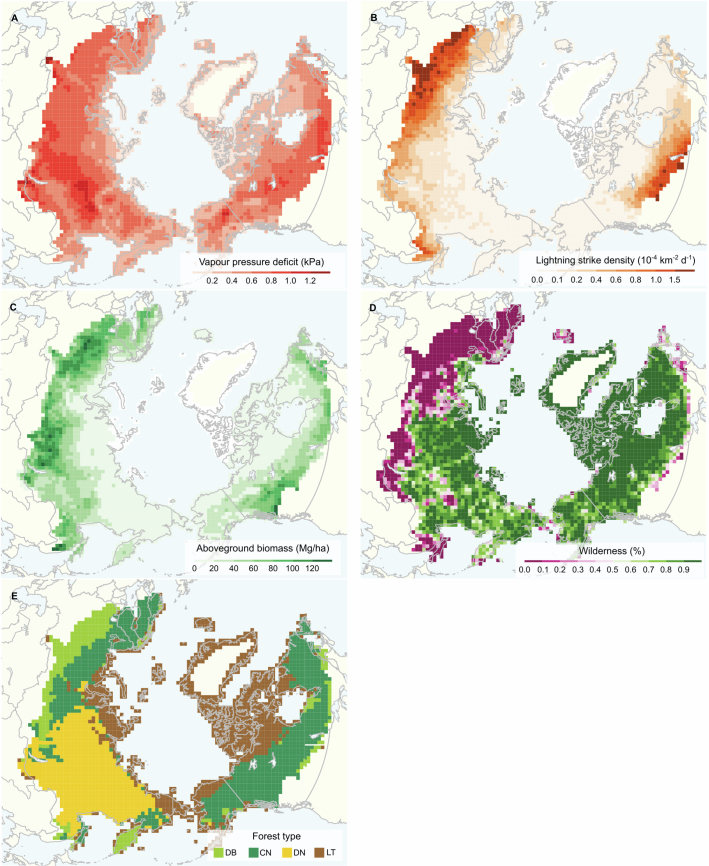
Maps of environmental drivers of fire activity. **a**, average vapour pressure deficit (VPD) in the month of maximum VPD. **b**, average lightning density between March and October. **c**, average aboveground biomass. **d**, fraction of pixel covered by wilderness according to the Human Footprint Map of 2013. **e**, dominant forest type for each pixel; DB: deciduous broadleaf, CN: coniferous evergreen needleleaf, DN: coniferous deciduous needleleaf, LT: low tree cover (<15%). Basemap: Natural Earth.

**Extended Data Fig. 4:**
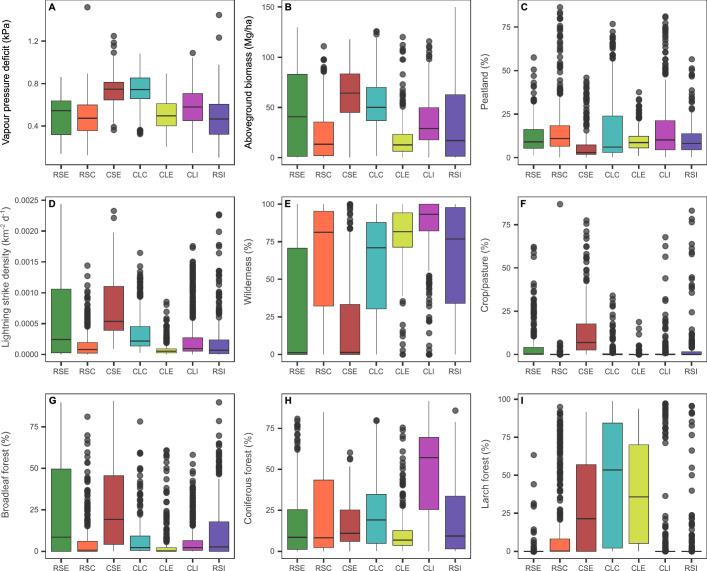
Environmental and climatic characteristics of the seven pyroregions. **A**, Vapour pressure deficit. **B**, Aboveground biomass. **C**, Peatland fraction. **D**, Lightning strike density. **E**, Fraction of wilderness areas. **F**, Cropland and pasture fraction. **G**, Fraction of broadleaf forest. **H**, Fraction of coniferous forest. **I**, Fraction of larch forest. Horizontal lines represent the median, and the lower and upper ends of each box are the 25th and 75th percentiles. Whiskers extend up to 1.5 times the interquartile range, sample points (grid cells) beyond that are represented as dots. RSE: rare-small-early (n = 328), RSC: rare-small-cool (n = 456), CSE: common-small-early (n = 185), CLC: common-large-cool (n = 394), CLE: common-large-early (n = 320), CLI: common-large-intense (n = 568), RSI: rare-small-intense (n = 297). See Fig. 3 for the geographical extent of the pyroregions.

**Extended Data Fig. 5:**
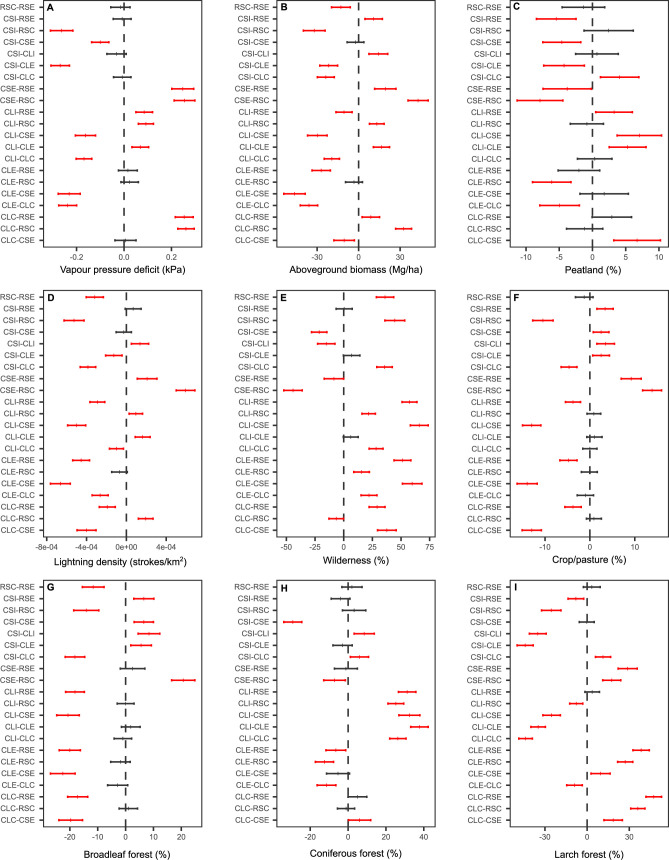
Results of Tukey Honest Significant Difference test of differences between pyroregions. **A**, Vapour pressure deficit. **B**, Aboveground biomass. **C**, Peatland fraction. **D**, Lightning strike density. **E**, Fraction of wilderness areas. **F**, Cropland and pasture fraction. **G**, Fraction of broadleaf forest. **H**, Fraction of coniferous forest. **I**, Fraction of larch forest. Shown are the mean difference (+) and 95% confidence interval (error bars). Red indicates significant differences between groups (alpha > 0.05). Black dashed line indicates no significant difference. RSE: rare-small-early (n = 328), RSC: rare-small-cool (n = 456), CSE: common-small-early (n = 185), CLC: common-large-cool (n = 394), CLE: common-large-early (n = 320), CLI: common-large-intense (n = 568), RSI: rare-small-intense (n = 297). See Fig. 3 for the geographical extent of the pyroregions.

The seven pyroregions exhibited significant differences in climate, human influence and vegetation properties (Extended Data Figs. 3–5). Pyroregions dominated by small, early-season fires (CSE and RSE) showed the largest anthropogenic impact, occurring in regions with low levels of wilderness area. In contrast, pyroregions dominated by large fires, such as CLE and CLI, occurred in regions with higher percentages of wilderness area. Pyroregions with a strong anthropogenic influence exhibited either exceptionally high fire number density (for example, in CSE) or low fire number density (for example, in RSE). The highest vapour pressure deficit (VPD) levels were observed in the CSE and CLC pyroregions located in the strongly continental climate zone of southern and central Siberia, followed by the CLI pyroregion in central and eastern boreal North America. Pyroregions with higher summer VPD in Eurasia (CSE and CLC) were also associated with higher above-ground biomass and lightning densities (Table 1 and Extended Data Fig. 4). The CLE, RSC and RSI pyroregions had the lowest above-ground biomass levels, suggesting that fuel limitations may restrict the fire activity in these clusters. The pyroregions showed a clear division according to tree species, with deciduous needle-leaved larch species dominating in the CSE, CLC and CLE pyroregions and evergreen needle-leaved conifers dominating in the CLI pyroregion (Extended Data Fig. 4). Mixed forests with a large fraction of broadleaved trees prevailed in the RSE and CSE pyroregions.

## Drivers of spatial variability in fire regimes

Distinct spatial patterns of fire activity in Arctic–boreal regions may be caused by spatial variations in climate and fire weather, differences in fuel load and structure, and the influence of humans on ignition and fire suppression. Our domain-wide grid-cell-based linear models, which used the multi-year average of VPD in the month of maximum VPD, lightning density, above-ground biomass, wilderness fraction and cropland and pasture fraction as predictor variables, explained 30%, 29% and 13% of the spatial variability in burned area, fire number and fire size, respectively (Extended Data Table 1). Using variables from the Canadian Fire Weather Index System^23^ instead of VPD yielded similar explanatory power in all models. Models incorporating tree species yielded slightly superior results overall compared with models without them, particularly in predicting fire radiative power (*R*^2^ = 0.30 versus *R*^2^ = 0.14; Extended Data Table 1). This observation aligns with the earlier research of ref. ^24^, which demonstrated that the presence of coniferous, fire-embracing tree species, such as black spruce (*Picea mariana*), which is prevalent in boreal North America, leads to higher fire intensity. By contrast, the presence of fire-resisting tree species such as larch (*Larix* sp.), which are common in northeastern Eurasia, leads to a lower fire intensity.

**Fig. 4:**
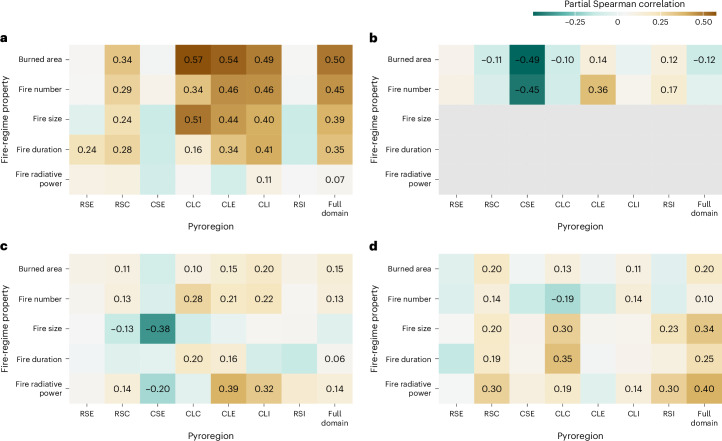
Spatial relationships between environmental variables and fire-regime properties in the seven Arctic–boreal pyroregions. Environmental variables include VPD (**a**), lightning density (**b**), above-ground biomass (**c**) and wilderness fraction (**d**). Relationships are expressed as partial Spearman correlation coefficients. Colours indicate the strength and direction of correlation. Values are given for all partial correlations with *P* < 0.05 (*P* values were computed using algorithm AS 89, two-sided, implemented in the cor.test R function). RSE, rare-small-early; RSC, rare-small-cool; CSE, common-small-early; CLC, common-large-cool; CLE, common-large-early; CLI, common-large-intense; RSI, rare-small-intense. See Fig. 3 for the geographical extents of the pyroregions. Source data

VPD is a strong driver of fire activity since it regulates fuel moisture and thus governs ignition efficiency and fire spread^25^. The multi-annual average maximum VPD was the strongest predictor for spatial patterns of burned area (partial Spearman correlation *ρ*_part_ = 0.50), fire number (*ρ*_part_ = 0.45), fire size (*ρ*_part_ = 0.39) and duration (*ρ*_part_ = 0.35) but not fire intensity, which may be linked with species-specific fire traits (Fig. 4a). Pyroregions with large fire sizes (CLC, CLE and CLI) showed the highest sensitivity to VPD, whereas RSE, RSI and CSI pyroregions were less sensitive to spatial variations in VPD. This suggests that anthropogenic activities, or a larger fraction of broadleaf deciduous forest, may attenuate the climate sensitivity of some boreal fire regimes.

Lightning and fire number were positively correlated in the CLE and RSI pyroregions (Fig. 4b), indicating that lightning occurrence may limit fire number in a quarter of the Arctic–boreal domain, including recent fire hotspots in eastern Siberia and western Alaska. Fuel moisture constraints may, however, be more important than strike density for initiating a fire start in large parts of boreal North America and central Siberia^26^. Regions with higher lightning density in southern Eurasia (Extended Data Fig. 3) displayed negative partial correlations between lightning and fire number. This counter-intuitive relationship may arise due to a dominance of anthropogenic ignitions (for example, in CSE, southern Siberia) or strong fire suppression (for example, in RSC, northern Europe^27,28^).

**Fig. 5:**
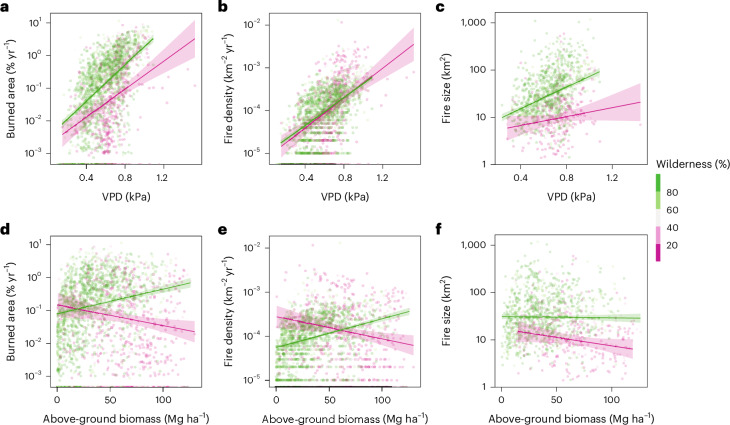
The dependence of fire-regime properties on climate and fuel are modulated by the fraction of wilderness. **a**–**f**, Scatter plots for vapour pressure deficit (VPD) (**a**–**c**) and above-ground biomass (**d**–**f**) versus burned area (**a**,**d**), fire number density (**b**,**e**) and fire size (**c**,**f**). All *y* axes are log-scaled. Lines and shaded areas refer to least-squares fit to the data and 95% confidence interval based on groups of 0–20% wild (pink) and 80–100% wild (green). Relationships of burned area and fire size with VPD are weaker in the presence of humans. Relationships between fire-regime properties and above-ground biomass reverse in the presence of humans. Panels **c**,**f** show only grid cells with at least 5 fires (*n* = 1,194); all other panels include data from all grid cells (*n* = 2,472).

The fraction of wilderness was the best single predictor of fire intensity (*ρ*_part_ = 0.40), and also correlated positively with fire size and duration across the entire domain (*ρ*_part_ = 0.34 and *ρ*_part_ = 0.25; Fig. 4d). Land use and anthropogenic activities further significantly modulated the influence of climate and fuel availability on the spatial distribution of fire properties (Fig. 5 and Extended Data Table 2). For example, in human-dominated regions with a low wilderness fraction, the sensitivity of fire size to VPD was lower than in more remote areas with a high wilderness fraction (Fig. 5c). The weaker VPD response in areas with a stronger human footprint may be a result of fire suppression and increased landscape fragmentation^29^. Indeed, while VPD was a better spatial predictor for fire size overall, 86% of grid cells with an average fire size larger than 100 km^2^ had a wilderness fraction greater than 50%.

Burned area, fire number and fire size also had diverging responses to fuel density in low and high wilderness areas (Fig. 5d–f), again highlighting the potential importance of fire suppression in human-dominated ecosystems. Positive relationships between fire-regime properties and above-ground biomass prevailed in pyroregions with a higher wilderness fraction (CLC, CLE, CLI and RSC). By contrast, the anthropogenically dominated CSR pyroregion exhibited negative correlations between above-ground biomass and fire size and intensity (Fig. 4c).

## Response of pyroregions to interannual climate variability

**Extended Data Fig. 6:**
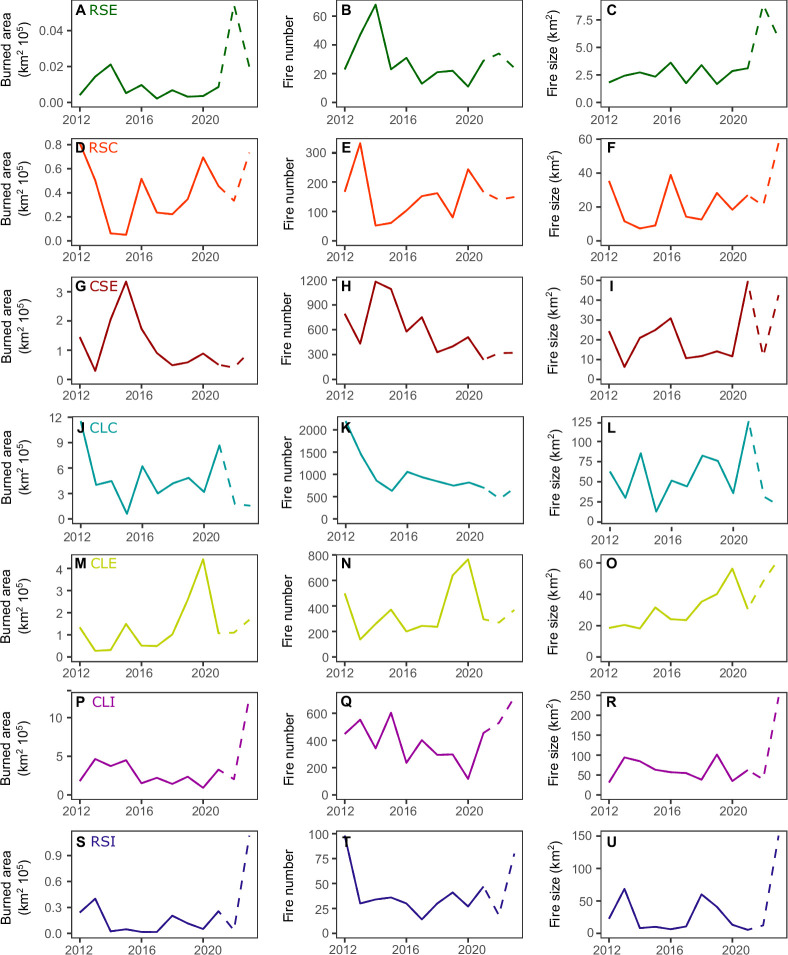
Annual burned area, number of fires and average fire size between 2012 and 2023 in the seven pyroregions. **A**, **D**, **G**, **J**, **M**, **P**, **S**: Total burned area. **B**, **E**, **H**, **K**, **N**, **Q**, **T**: Fire number. **C**, **F**, **I**, **L**, **O**, **R**, **U**: Fire size. Dashed lines refer to data that is based on Visible Infrared Imaging Radiometer Suite near real-time active fire locations instead of the science quality archive active fire product. RSE (**A**–**C**): rare-small-early, RSC (**D**–**F**): rare-small-cool, CSE (**G**–**I**): common-small-early, CLC (**J**–**L**): common-large-cool, CLE (**M**–**O**): common-large-early, CLI (**P**–**R**): common-large-intense, RSI (**S**–**U**): rare-small-intense. See Fig. 3 for the geographical extent of the pyroregions.

The specific environmental conditions that shape fire regimes in different pyroregions may also strongly modulate the sensitivity of fire activity to interannual variation and long-term trends in climate. We therefore investigated the interannual correlation of fire-regime properties with summer VPD and fire weather index variables within each pyroregion to assess which pyroregions were most sensitive to climatic variations. Burned area, fire number and fire size were most sensitive to fire-prone weather in pyroregions with large fire sizes, particularly in Siberia (Extended Data Table 3). Fire activity in the RSC pyroregion located largely in Northern tundra regions was sensitive to fire weather to a lesser degree. Fire activity and, in particular, fire sizes in the RSE, RSI and CSE pyroregions were least sensitive to VPD. This indicates that interannual variations in fire weather drive ignitions and spread especially in remote pyroregions. Pyroregions that experienced fire extremes in recent years, such as central and eastern Siberia and large swaths of Canada (Extended Data Fig. 6), showed a strong climate sensitivity.

## Implications for future Arctic–boreal fire activity

Arctic–boreal regions are warming nearly four times faster than the rest of Earth^30^, and fire activity is projected to increase due to associated decreases in fuel moisture^31^ and increases in lightning ignitions^13,26^. An intensification of regional fire regimes has already been observed within the Arctic Circle^2^ and in parts of Canada^7,32,33^ and Alaska^34^. In this Article, we show that the sensitivity of fire activity to a warmer and drier climate varies substantially between pyroregions. In line with observed emerging trends, we found that fire activity is most sensitive to climate in boreal North America, eastern Siberia and northern tundra regions. Conversely, regions in southern Siberia and Europe may be more resilient to increases in heatwaves and droughts, since a larger human footprint in these areas may contribute to more fragmented fuels and more effective fire suppression.

Projected increases in Arctic–boreal lightning activity with climate warming^13^ are particularly important in driving future increases of fire activity in western Alaska and eastern Siberia, where fire number was sensitive to lightning strike density. Notably, these regions, sensitive to lightning, have already experienced recent fire extremes^2–4^, underscoring the potential impact of lightning-caused fire complexes on annual burned area. Furthermore, rising temperatures may enhance ignition efficiency in currently moisture-limited pyroregions, heightening their susceptibility to concurrent increases in lightning^26^.

In parallel with the Arctic–boreal regions transitioning into a warmer climate with an increasing likelihood of compounding extremes^35^, abrupt biome shifts have been observed^36,37^ and projected^38,39^, with major implications for fire regimes. Some biome shifts may exert a positive feedback on fire activity, such as forest transitions from more open and older stands to denser and younger stands in Siberian larch forests^39,40^, shrub expansion in tundra areas^41,42^ and tree-line shifts^43,44^. Negative feedbacks can emerge through forest transitioning from flammable conifers to less flammable deciduous forests^45–48^, regeneration failures^49–51^ or decadal self-limitation of fire occurrence and spread^52^. While fuels were generally less influential than climate in shaping spatial patterns of fire activity, we found that fuel load was an equally important driver of fire intensity and burned area in more remote pyroregions. Fuel type also governed fire activity, with pyroregions with higher fractions of deciduous broadleaf forests displaying lower fire activity.

Furthermore, while the effects of climate warming and fires on above-ground biomass have been extensively studied, the sensitivity of below-ground peat and permafrost carbon pools to these changes remains poorly understood^53,54^. Arctic–boreal regions contain extensive peatlands, such as the western Siberian lowlands and the Hudson plains. These peatlands currently experience relatively limited fire activity. Thus, boreal peatlands may be relatively resistant to fires under current climate and permafrost conditions due to hydrologic self-regulation^54–56^. However, data on the presence and hydraulic state of peatlands are scarce in many Arctic–boreal regions, hindering predictions about the sensitivity of peatland burning to climate warming and associated permafrost degradation and ecosystem shifts^53^.

Continued expansion of agriculture, logging and resource extraction and wildland–urban interfaces^57,58^ increases human vulnerability to fire but may also strongly influence fire regimes^59^. Anthropogenic activities influence fire activity directly, through intentional or unintentional ignition and fire suppression, and indirectly through fuel management, logging and fragmentation^60,61^. Human activities also increase peatland vulnerability to fires through land-use changes that enhance drainage and degrade peatlands^54,55^. While many anthropogenic activities currently suppress fire activity, the combination of a warming climate and long-term fire prevention practices in many populated boreal regions may increase the risk of escaped fires in vulnerable areas^61^. Ongoing efforts to better represent fire-suppressing and fire-inducing effects of anthropogenic presence in fire models are therefore critical to improving the representation of boreal fires^62,63^. Understanding the interplay between fuels, climate and ignition sources and their varying importance in different pyroregions is vital for improving future predictions of changing Arctic–boreal fire regimes. Our analysis uniquely identifies these interconnected drivers of spatial variability in fire regimes. It shows that some Arctic–boreal pyroregions, particularly those that have experienced recent fire extremes, exhibit a strong climate sensitivity. By contrast, fire activity in pyroregions of southern Siberia and northern Europe may have a lower sensitivity, probably as a partial consequence of greater landscape fragmentation caused by human activity. In future work, extending the fire tracking and classification system we developed here to temperate and tropical regions may help to identify additional vulnerable regions, especially in areas lacking reliable long-term fire records.

## Methods

### Fire data

We used data from the Arctic–boreal fire atlas based on active fire location products from the VIIRS aboard the Suomi National Polar-orbiting Partnership satellite. This dataset was generated by tracking individual fires in the study area between 2012 and 2023 at 12 hourly time steps. A variety of fire properties, including daily perimeters, daily size, the current fuel type, fire spread, fire line length and fire intensity (fire radiative power at the active fire line) were recorded for each time step. A full description of the fire tracking algorithm and dataset, as well as the validation of the dataset, can be found in the Supplementary Information.

### Study area

To delineate the Arctic–boreal biomes, we used the World Wildlife Fund terrestrial ecoregions^64^ shapefile. We selected all ecoregions corresponding to the ‘Boreal Forests/Taiga’ and ‘Tundra’ biomes and applied a 0.5° buffer to this region so that fires at the border of our study area would not be cut off abruptly when spreading over the biome boundaries. During postprocessing, only fires that intersected with the study domain were retained.

### Selection of fires for the analysis

Arctic–boreal regions comprise a variety of fire types, many of them being small in size and short in duration. The VIIRS active fire product can pick up fires as small as a bonfire^65^, and many of the recorded small and short-lived fires in our database were anthropogenically caused fires such as agricultural fires. Furthermore, many unmapped refineries and other gas flaring areas, for example, in western Siberia, were falsely identified as vegetation fires in the VIIRS product. While all of these were retained in the fire atlas product, we applied a stricter filtering routine to the database used for the characterization of Arctic–boreal pyroregions here since the large number of very small fires added very little burned area but had a large effect on the statistics of, for example, fire number and size.

In total, we recorded 104,973 Arctic–boreal fires, which burned an area of 1.2 million km^2^ between 2012 and 2021. While 63% of the recorded fires were smaller than 1 km^2^, the 10% largest fires accounted for 91% of the total burned area. As a first step, we therefore filtered out small agricultural fires for fires whose final fire type was classified as Cropland, Grassland, Urban or Other. We also excluded fires that started in January, February, November or December. These two filters were tailored to filter out agricultural burns and other small fires caused by people such as campfires, trash burning or bonfires. As a next step, we applied a series of filters aimed at filtering out gas flares. Due to spatial lumping of production areas and refineries, fire detections associated with gas flaring were not always constricted to single VIIRS pixels. However, true wildfires can also burn close to areas of known gas flaring. To find suitable fire size thresholds, we therefore used a database of known gas flaring sites^66^. We selected all fires smaller than 20 km^2^ that overlapped with at least one known gas flare and built a model predicting the fire size from the number of gas flare sites overlapping with each fire. We then used the upper prediction interval as a cut-off for the fire size. The cut-offs ranged from 2.5 km^2^ for one gas flare overlap to 21 km^2^ for fires with 25 overlapping gas flare sites. We then removed only fires that were smaller than the computed cut-off value based on the number of gas flare overlaps. Since the known gas flaring record was not exhaustive, we further applied two additional filters on the fires smaller than 20 km^2^. The first of these filters included a search for very long-lasting fires (>100 days) with little daily fire spread (<1 km^2^ d^–1^). Further, we searched for overlapping fires (<20 km^2^) between 2012 and 2021 and removed fires from the record if they overlapped in more than 2 years. This procedure also removed repeated agricultural burns that were not previously detected. Last, we filtered out all fires with a final size of less than 1 km^2^ as these also most likely represented agricultural or other human-caused fires and added little to the total burned area. Using the fire identification numbers of the final set of filtered fires, we also kept only the ignitions associated with these fires in a filtered ignition database. Our final selection comprised 26,504 fires that burned an area of 1.12 million km^2^.

### Mapping of fire-regime properties and clustering into pyroregions

To derive fire-regime properties, we aggregated data from the filtered fires into a 100 km grid in a polar projected coordinate system (EPSG 3571). For the grid-cell statistics (fire density, fire size, fire duration, fire radiative power and fire start date), we computed two versions: one based on all fires whose centroid was located within a 250 km radius from the grid-cell centroid, and a second based on all fires that had a minimum overlap of 30% of their area with a grid cell. To aggregate the total burned area, we summarized the area of each fire that fell within a radius or grid cell, respectively. We used the first (radius-based) estimate for the maps and clustering into pyroregions, and we used the second estimate for the modelling part, to have an exact overlap between the fire data and environmental and climatic drivers. To compute the fire number and fire density, we aggregated the number of initial fire starts, irrespective of whether these would later merge into fire complexes. The annual percentage burned area displayed in Table 1 and Extended Data Fig. 2 uses burnable land areas as a baseline, excluding water and barren regions according to the 300 m European Space Agency Climate Change Initiative (ESA CCI) land-cover dataset used in the fire tracking system.

We used a model-based clustering approach to identify pyroregions with similar fire-regime characteristics using the six predictor variables shown in Fig. 2. The analysis was performed using the R Mclust package^67^, which deploys a finite Gaussian mixture modelling framework and enables the optimization of cluster sizes and shapes on the basis of the Bayesian information criterion (BIC). We first performed several modelling runs with different cluster shapes and cluster sizes ranging from one to nine, and selected a model allowing for a variable volume, shape and orientation of clusters, based on the highest BIC (Supplementary Fig. 3). While the BIC continued to increase with cluster numbers up to nine clusters, we chose a cluster number of seven, because the increase became negligible beyond that, following the methodology applied in ref. ^8^. Uncertainties were largely uniform across clusters and rarely exceeded 0.2 (85th percentile; Supplementary Table 4). Furthermore, clusters were mostly spatially contiguous, and cluster uncertainty was largest at the spatial borders between different clusters (Fig. 3), which speaks for the reliability of the cluster patterns.

For naming the pyroregions, we used the fire-regime properties that showed largest variability between clusters. To determine common versus rare fire frequency, we used a fire return interval threshold of 500 years, which was computed as the inverse of the annual percentage burned area displayed in Table 1. Reference ^50^ also used this threshold for classifying fire return intervals across Siberia. For fire size, we used a threshold of 40 km^2^ for large fires. Although the threshold for large fires is not explicitly stated in ref. ^8^, this cut-off aligns with their classification of large versus small fire sizes. For the classification of early versus late fires, we used a cut-off date of 30 June, and for the classification into cool and intense fires, we used a threshold of 100 W m^–2^. We grouped intensity and fire start timing together to balance between using the best descriptors for each pyroregion and having a simplistic (three-letter) classification.

To assess the influence of spatially varying overpass timings on fire radiative power estimates derived for the different pyroregions, we analysed the original VIIRS active fire locations associated with each fire used for characterizing the pyroregions. Histograms of overpass times (Supplementary Fig. 4a–g) reveal similar distributions across pyroregions. While the bimodal peaks of daytime and night-time overpasses were generally aligned for all pyroregions, the spread of overpass times was wider for regions located farther North due to an increasing overlap of overpasses. However, the main signal of fire radiative power differences between pyroregions (Table 1 and Extended Data Fig. 2) followed from the fire radiative power differences during peak daytime hours (11:00 to 14:00 lt; Supplementary Fig. 4h) and is therefore robust between pyroregions.

### Data for assessing drivers of spatial variability

Fuel availability, fuel moisture content and ignition sources are the three determining factors for fire occurrence in any ecosystem. In boreal regions, fire activity is limited mainly by an absence of ignition sources and a surplus of moisture, and fuel limitations can be particularly important in sparsely vegetated Arctic regions^10^. We chose a set of climatic and geographic variables to represent these three factors for assessing the drivers of spatial variability in fire activity (Extended Data Fig. 3). All data were reprojected to match the coordinate system of the fire maps and aggregated to their 100 km spatial resolution by computing the average of all overlapping pixels.

#### Climate

As a main proxy for fire weather influences on fuel moisture, we used the VPD, which has shown to be a suitable predictor of fuel flammability and thus fire ignitions and fire spread^25^. We computed VPD from ERA5-Land (fifth-generation European Centre for Medium-Range Weather Forecasts reanalysis) temperature and dewpoint temperature at 2 m as:

$${\rm{VPD}}={\rm{SVP}}-{\rm{AVP}}$$

Where the saturation vapour pressure (SVP) and the actual vapour pressure (AVP) were computed according to the Tetens equation:

$${\rm{SVP}}=\,0.61078\,\times {{\rm{e}}}^{\frac{(17.27\times {{T}})}{(237.15+{{T}})}}$$

where *T* is the temperature in degrees Celsius. For the computation of actual vapour pressure, the temperature is simply replaced by the dewpoint temperature in this equation. ERA5-Land products are delivered at a native resolution of approximately 9 km. As a proxy for the climatological fire danger, we identified the month with the maximum VPD for each pixel and computed a multi-year average of the average VPD during this month for the years 2012–2021.

We further used precomputed fire danger indices of the Canadian Fire Weather Index system provided by the Copernicus Emergency Management Service^68^. We computed monthly means for each 100 km grid cell from the daily reanalysis data at 0.25° resolution. The chosen variables were the Fine Fuel Moisture Code, which represents an approximation of the moisture content of fine fuels and indicates ignition potential, Duff Moisture Code, which represents the moisture content of the duff layer, and the Fire Weather Index, which represents an overall rating of fire danger, taking the moisture state of fine, medium and larger fuels into account. These variables are most commonly used for fire danger assessment but rely on accurate estimates of seasonal precipitation, wind speed and snow water equivalent, observations and validation of which are sparse in many remote Arctic and boreal regions^69^. We have therefore included these variables in alternative versions of our climate analysis but focus on VPD when reporting main results.

#### Lightning ignition

We extracted lightning density from the World Wide Lightning Location Network for the years 2012–2021^70^. This network consists of a number of radio receivers in the very-low frequency range (3–30 kHz) distributed throughout the world, which enable very long-range (thousands of kilometres) lightning detection with a spatial accuracy of about 5 km^71–73^. While detection efficiency is not uniform over the boreal regions due to gaps in the network in Siberia^72^, this dataset is, to our knowledge, the only currently openly available ground-based lightning detection network with global coverage. The data are distributed as a gridded, monthly aggregated climatology product at 5 arcmin resolution, which has been pre-processed to correct for the differences in the detection efficiency using gridded detection efficiency maps. We aggregated these data for the months March to October, the extended Arctic–boreal fire season, over all years to derive a climatological value that matches the temporal extent of the fire data.

#### Fuels

As a proxy for above-ground fuels, we used the GlobBiomass product^74,75^, a static above-ground biomass map of the year 2010. We further used the peatland map derived from the Northern Circumpolar Soil Carbon Database^76^ that was used in the fire tracking algorithm to derive the fraction of land covered by peatlands within each map pixel. Last, we derived the tree species dominance for each pixel on the basis of the ESA CCI land-cover data. We extracted annual tree cover per forest type (deciduous broad-leaved, coniferous needle-leaved and deciduous needle-leaved) following ref. ^77^ and computed the average percentage of the three forest types for each pixel. For the tree species analysis, we used data from 2020 since large-scale tree species abundance changed little throughout 2012–2020.

#### Human impact

Humans shape fire regimes through their impact on ignitions and active fire suppression, but also through their influence on fuel continuity and fuel type. We included two datasets to account for different types of human impact. We used the History Database of the Global Environment (HYDE version 3.2.1)^78^ for estimating the percentage of croplands and grazing lands in each pixel. HYDE is provided at a 5 arcmin resolution and contains data for the Common Era from 2012 to 2015. To estimate the percentage of each pixel that was completely untouched by humans, we used the Human Footprint Map of the year 2013^79,80^. The Human Footprint Maps approximate the magnitude of human influence on a global map using data on build-up areas, population density, infrastructure elements and crop and pasture lands and are delivered at a spatial resolution of 1 km^2^. A value smaller than four in these maps has previously been used to identify wilderness areas with no human influence^79^. To be conservative, we classified only pixels with a value of zero as wilderness. Thanks to the higher spatial resolution of the human footprint maps compared with the grid-cell size in our analysis, we calculated the wilderness fraction of each grid cell by computing the percentage of all 1 km^2^ pixels containing wilderness within each 100 km× 100 km pixel.

### Drivers of spatial variability and climate sensitivity

As a first step to assessing drivers of spatial variability in fire activity, we investigated differences in the geographic and climatic drivers between the clusters using box plots (Extended Data Fig. 4) and Tukey’s honest significant difference tests to assess significant differences between pyroregions (Extended Data Fig. 5). We further computed the partial Spearman correlation between all drivers and fire-regime characteristics within pyroregions and across the Arctic–boreal biomes. Last, we built linear models predicting fire-regime characteristics from the environmental and climatic driver to assess the overall predictability of fire activity within and across pyroregions.

The sensitivity of the pyroregions to climate (Extended Data Table 3) was assessed by computing grid-cell-based Spearman correlations between annual burned area, fire density and fire sizes, and the annual average of VPD in the month of maximum VPD. For each pyroregion, we reported the average and standard deviation of the correlations of all grid cells that contained at least 5 fires and at least 3 years of data.

## Online content

Any methods, additional references, Nature Portfolio reporting summaries, source data, extended data, supplementary information, acknowledgements, peer review information; details of author contributions and competing interests; and statements of data and code availability are available at 10.1038/s41561-024-01505-2.

## Supplementary information

Supplementary Information Supplementary Figs. 1–4, Tables 1–4 and Methods.

## Source data

Source Data Fig. 4 Partial correlation coefficients and *P* values for all comparisons shown in Fig. 4.

## Figures and Tables

**Extended Data Table 1:**
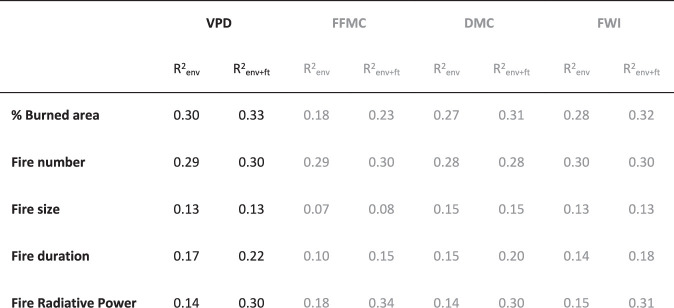
Model coefficient of determination (R2) for linear models predicting the spatial pattern of fire characteristics from environmental variables and a climate variable

Environmental variables: lightning density, wilderness fraction, aboveground biomass, fraction of croplands and pastures. Climate variable: one of vapor pressure deficit (VPD), fire weather index (FWI), fine fuel moisture code (FFMC), or duff moisture code (DMC). R^2^_env_: models using only environmental/climate variables. R^2^_env+ft_: models including environmental/climate variables and the fraction of forest type (deciduous-broadleaf, deciduous-needleleaf, and coniferous evergreen needleleaf). Only grid cells with at least five fires (n = 1120) were used for the models to assure representative values for average fire size, duration and fire radiative power.

**Extended Data Table 2:**
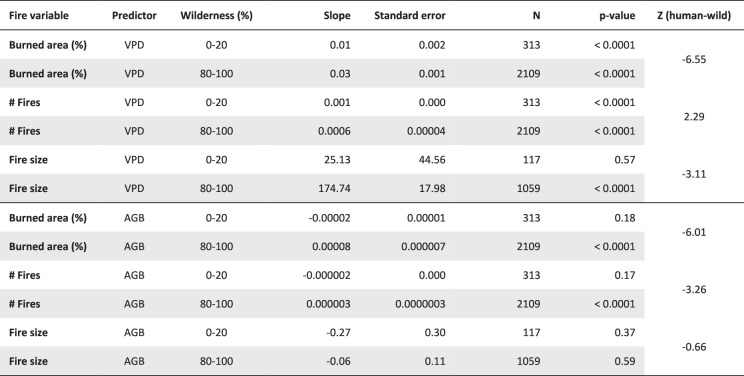
Relationships between fire-regime properties and vapor pressure deficit and aboveground biomass are modulated by the fraction of wilderness

Mean and standard error of the slopes for human-dominated (0-20% wilderness) and wild (80-100% wilderness, shaded gray) areas were tested with a two-sided t-test. Z scores were computed from the mean and standard error of the two wilderness groups. Regressions for fire size were computed based on grid cells with at least 5 fires, all other regressions are based on all grid cells.

**Extended Data Table 3:**
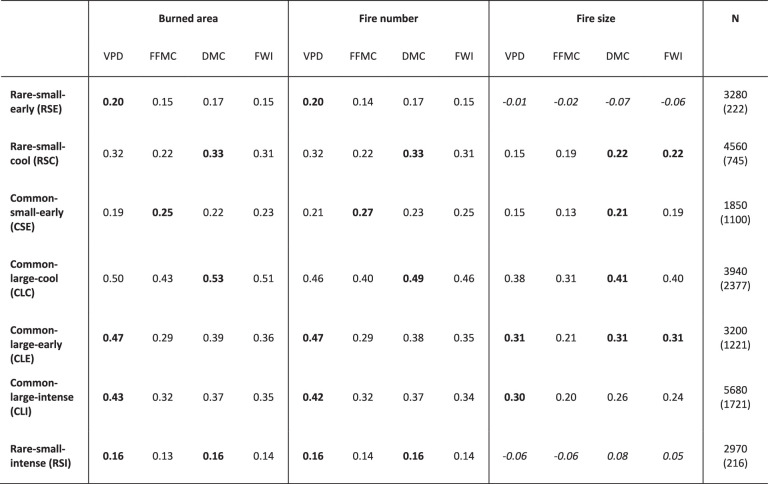
Spearman correlation between annual fire activity and fire weather indicators for the seven pyroregions

Fire weather values represent averages for the month of maximum vapor pressure deficit for each grid cell. Each annual observation for each grid cell serves as one observation. N refers to the total number of observations (grid cells x years). The number of grid cells in parentheses is the number of grid cells with at least one fire used for the fire size correlation estimation. Values printed in italics are not significant (p > 0.05). Values printed in bold refer to the fire weather variable with the highest correlation per pyroregion and fire variable. VPD: Vapor pressure deficit, FWI: Canadian Fire Weather Index, FFMC: Fine Fuel Moisture Code, DMC: Duff Moisture Code.

## Data Availability

All data used for this research are freely available. The Arctic–boreal fire atlas data from 2012 to 2023 can be accessed via Pangaea (10.1594/PANGAEA.967653). VIIRS active fire locations can be downloaded from the University of Maryland (https://modis-fire.umd.edu) and NASA’s Fire Information for Resource Management System (https://firms.modaps.eosdis.nasa.gov/). ERA5 reanalysis data can be retrieved from the Copernicus Climate Data Store (https://cds.climate.copernicus.eu). The GlobBiomass data can be found via Pangaea (10.1594/PANGAEA.894711). Lightning density form the World Wide Lightning Location Network can be found at Zenodo (https://zenodo.org/records/6007052) (ref. ^81^). Human Footprint Maps can be downloaded from UNEP-GRID-Geneva (https://datacore-gn.unepgrid.ch/geonetwork/srv/api/records/a967c8b4-3169-4848-a624-f14946b53a24). Source data are provided with this paper.

## Code availability

The fire tracking code used for the Arctic–boreal fire atlas is freely accessible via Zenodo (10.5281/zenodo.10611948) (ref. ^22^).
