# Supplementary material for: Spatial variability in Arctic–boreal fire regimes influenced by environmental and human factors
Source: Nat Geosci. 2024 Sep 2;17(9):866–73. doi: 10.1038/s41561-024-01505-2 (PMC11387193; doi:10.1038/s41561-024-01505-2)
Supplement: Supplementary file 1 — Supplementary Figs. 1–4, Tables 1–4 and Methods. [file 41561_2024_1505_MOESM1_ESM.pdf]

nature geoscience

Supplementary information

https://doi.org/10.1038/s41561-024-01505-2

Spatial variability in Arctic–boreal fire regimes influenced by environmental and human factors

In the format provided by the authors and unedited

Supplementary text: Fire tracking algorithm description and validation

Fire atlas data

We used the active fire location products (VNP14IMGML, Collection 1 Version 5) from the Visible Infrared Imaging Radiometer Suite (VIIRS) aboard the Suomi National Polar-orbiting Partnership (Suomi-NPP) satellite for tracking all individual fires in the study area between 2012 and 2023. While including data from the VIIRS NOAA-20 satellite may have allowed for tracking at higher temporal resolution in high-latitude regions, NOAA-20 was only launched in 2018 and was therefore excluded from our analysis to provide a temporally consistent time series over a longer duration interval. VIIRS has a minimum of two daily overpasses with local overpass times at 1:30 am and 1:30 pm for each location on Earth with an increasing overpass frequency at high latitudes due to overlapping scan tracks. Due to its higher spatial resolution, VIIRS has shown to increase burned area detections in boreal regions by more than 40 % compared to its predecessor, the Moderate Resolution Imaging Spectroradiometer (MODIS)[1]. For 2012 to 2021, science-quality VNP14IMGML active fire locations were available from the University of Maryland VIIRS Active Fire website (https://viirsfire.geog.umd.edu/). We used only active fire detections with nominal or high confidence, and those flagged as vegetation or offshore fires. Offshore fires were included since vegetation fire detections in lake-rich areas such as Northern Canada or northeastern Siberia were frequently erroneously flagged as offshore fires in the active fire product. For 2022 and 2023 we used VIIRS active fire locations from NASA's Fire Information for Resource Management System (FIRMS, https://firms.modaps.eosdis.nasa.gov/). For 2023, this included data up to October 31, 2023. These real-time fire locations were not used for the clustering into pyroregions, but are provided in the published dataset and were used to extend the time series in Extended Data Figures 1 and 6 (dashed lines). To eliminate the numerous gas flaring fires that were

erroneously flagged as vegetation fires in the VIIRS active fire product we filtered out fire locations that overlapped with known gas flare locations according to the NOAA gas flare dataset[2].

To assemble active fire detections into fire clusters, we determined spatial and temporal thresholds according to land cover and peat presence underlying the fire locations of each new time step. To determine the dominant land cover, we used the annual European Space Agency (ESA) Climate Change Initiative (CCI) land cover dataset at 300m resolution from the EC C3S Land cover project. We used version 2.0.7 for the years 2012 – 2015, and version 2.1.1 for all other years. Since land cover data was not yet available for the years 2021 to 2023, we used 2020 data in replacement. The land cover data was aggregated into six classes (forest, shrubland/mosaic/open, grassland, cropland, urban and other) using a conversion table (Supplementary Table 1). In addition, we used the maps of northern peatland extent version 2 from the Bolin Centre for Climate research[3] to assess peat presence. We flagged a 10km pixel (and each overlapping fire location) as potential peatland if combined fraction of Histel (perennially frozen organic soil) and Histosol was larger than 10 %.

To minimize commission errors we corrected all fire perimeters using high-resolution lake perimeters derived during postprocessing. We used the annual Global Surface Water dataset version 5 provided by the Joint Research Center from 2012 to 2021[4] to create an annual inventory of permanent surface water features. The original 0.00025 degree (approximately 30m) resolution data was gridded to a projected polar coordinate system (EPSG 3571) at 90m resolution using nearest neighbor interpolation to reduce the processing time for feature extraction. Individual lakes were extracted from this grid using the same alpha hull algorithm

that was used for the fire tracking (see section 'Fire tracking algorithm'), using an alpha of 100 m to account for the higher spatial resolution of the lake data.

Fire tracking algorithm

We adapted the fire object tracking system deployed in the Californian Fire Events Database[5] for Arctic-boreal fires. In a first step, the fire locations of each day were clustered into 12-hour time steps using the local solar time, with local solar times between 6am and 6pm being classified as day time overpasses, and those between 6pm and 6am as night time. It is important to note that in the case of high latitudes the day/night flag of VIIRS active fires cannot be accurately used for differentiating between morning and afternoon overpasses since it relies on solar zenith angles. The fire locations were transformed to a projected polar coordinate system (North Pole Lambert Azimuthal Equal Area, EPSG 3571) so that distance calculations and buffering operations are accurately performed throughout the fire tracking procedure.

The locations of each time step were clustered using a ball tree algorithm and a query radius of 1000 m. To assess whether a cluster belonged to an existing fire, each cluster was compared with all existing active fires using an rtree[6] (Python ctypes wrapper (http://toblerity.org/rtree/)) of libspatialindex (https://libspatialindex.github.io/) neighbor search. The search radius was adjusted to the dominant land cover of the last active fire detections of each fire according to Supplementary Table 2. Likewise the maximum time of inactivity was set based on the land cover and peat presence of the last active fire detections. Boreal fires can smolder for extended periods of time[7]. To account for this, we implemented a 'sleeper' procedure, which allowed fires to stay active for up to 30 days at locations along an active fire line located in forests or areas with peat. These 'sleeper' fires were allowed to reactivate when new fire pixels were detected within 1000 m of the previous active fire line.

As a final step, the algorithm merges fire complexes that have grown together with the inclusion of new fire clusters using the same thresholds in Supplementary Table 2.

Computing fire attributes

To create the fire geometries at each time step, we used the alpha shape algorithm which has been successfully applied in previous work to represent fire perimeters[5,8]. In the alpha shape, a concave hull of a set of points, or fire pixels, is computed using a disk with a fixed radius that it fitted piecewise to form the edges of the polygon. The alpha parameter defines the radius of the disk and thereby how tightly the hull is fitted around the points. Following Chen et al.[5], we used an alpha of 1000 m, which represented a good compromise between capturing fine-scale fire features and retaining the coherence of the contiguous fire perimeters. An example of all half-daily perimeters created for a large fire in Siberia with multiple ignitions is presented in Fig. 1B.

Apart from the geometries for each time step, the fire tracking algorithm records a variety of fire properties over time, such as daily size, the current fuel type, fire spread, fire line length and fire intensity. In this study, we used only a part of the complete suite of statistics, including the ignition timing and locations, final fire perimeters and fire sizes, total duration of fires and fire radiative power. Sub-daily estimates of fire radiative power (FRP) were derived by averaging the pixel-normalized FRP values at the fire line per fire and time step. As a metric of intensity over the lifetime of a fire, we computed the 95th percentile of fire intensity per fire based on these sub-daily fire radiative power statistics.

At the end of their lifetime, wildfires may smolder for extended periods of time without transitioning to flaming again. These smoldering hotspots can be picked up by VIIRS, leading to elongated fire durations during which no fire spread is occurring. To account for this

residual smoldering, we used the time series of sub-daily fire size estimates for each fire to compute a corrected duration. As a corrected extinction time we thereby used the first detection when 95 percent of the final fire size was reached.

We generated annual vector layers of all final fire perimeters and ignition locations. The final fire type was derived using the dominant land cover type over the whole fire scar based on a combination of ESA CCI land cover data and the circumpolar Arctic vegetation map[9]. For each fire, the class with the largest overlap was chosen as dominant land cover. Fire perimeters that fell within tundra vegetation according to the circumpolar Arctic vegetation map were classified as tundra fires. For all other fires, we used the classes delineated in Supplementary Table 1.

Validation of the fire atlas

We validated the final perimeters from our fire atlas using governmental fire perimeter data from the Alaskan and Canadian fire databases. For Alaska, we used the Alaska Wildland Fire Maps (AWFM, https://fire.ak.blm.gov/predsvcs/maps.php), and for Canada the National Burned Area Composite (NBAC) developed by the Canada Centre for Mapping and Earth Observation and the Canadian Forest Service[10]. Official fire perimeter databases often rely on higher-resolution imagery (i.e. from Landsat) than the VIIRS active fire locations we used, and may therefore contain more accurate spatial information. Nevertheless, the interannual burned area and number of fires from our fire atlas showed a close agreement with the governmental databases (Supplementary Fig. 1). The largest disparities were seen for the total number of fires in Canada, mainly due to small agricultural burns in southern Manitoba, Saskatchewan and Alberta, which were not mapped in NBAC since they did not exceed the minimum mapping unit. The official perimeter data slightly differed between Alaska and Canada, with NBAC accounting for unburned islands within perimeters, whereas AWFM only

contain outer perimeters. The VIIRS-generated perimeters developed here contain unburned islands, but exclude lakes, which may explain the slightly lower burned area compared to, and larger burned area compared to NBAC resulting for example in the lake-rich Northwest Territories (Supplementary Fig. 2, Supplementary Table 3). For the fires detected by both products, fire sizes were generally similar, with largest deviations for fires that were erroneously merged or split up in too many parts due to missing fire detections (Supplementary Fig. 2).

References

1. Xu, W., Scholten, R. C., Hessilt, T. D., Liu, Y. & Veraverbeke, S. Overwintering fires rising in eastern Siberia. *Environ. Res. Lett.* **17**, 045005 (2022).

2. Elvidge, C. D., Zhizhin, M., Baugh, K., Hsu, F. C. & Ghosh, T. Methods for global survey of natural gas flaring from visible infrared imaging radiometer suite data. *Energies* **9**, (2016).

3. Hugelius, G. *et al. Maps of northern peatland extent, depth, carbon storage and nitrogen storage*. https://doi.org/10.17043/hugelius-2020-peatland-2 (2021) doi:10.17043/hugelius-2020-peatland-2.

4. Pekel, J. F., Cottam, A., Gorelick, N. & Belward, A. S. High-resolution mapping of global surface water and its long-term changes. *Nature* **540**, 418–422 (2016).

5. Chen, Y. *et al.* California wildfire spread derived using VIIRS satellite observations and an object-based tracking system. *Sci. Data* **9**, 249 (2022).

6. Guttman, A. R-trees. *ACM SIGMOD Rec.* **14**, 47–57 (1984).

7. Scholten, R. C., Jandt, R., Miller, E. A., Rogers, B. M. & Veraverbeke, S. Overwintering fires in boreal forests. *Nature* **593**, 399–404 (2021).

8. Hantson, S., Andela, N., Goulden, M. L. & Randerson, J. T. Human-ignited fires result in more extreme fire behavior and ecosystem impacts. *Nat. Commun.* **13**, 1–8 (2022).

9. Walker, D. A. *et al.* The Circumpolar Arctic Vegetation Map. *J. Veg. Sci.* **16**, 267–282 (2005).

10. Hall, R. J. *et al.* Generating annual estimates of forest fire disturbance in Canada: The National Burned Area Composite. *Int. J. Wildl. Fire* **29**, 878–891 (2020).

Supplementary Table 1: **Conversion table for simplifying the land cover classes of the European Space Agency Climate Change Initiative Land cover product.**

New land cover class	Original land cover classes
Other	0 (no data), 200 (bare areas), 210 (water bodies), 220 (permanent snow & ice)
Cropland	10 (rainfed cropland), 20 (irrigated cropland), 30 (mosaic cropland/natural vegetation), 40 (mosaic natural vegetation/cropland)
Forest/Tree cover	50 (broadleaved >15%), 60 (deciduous > 15%), 70 (evergreen needleleaved > 15%), 80 (deciduous needleleaved > 15%), 90 (mixed leaf type), 160 (flooded, fresh water), 170 (flooded, saline)
Shrubland/ mosaic/ open	100 (mosaic tree and shrub/herbaceous), 110 (mosaic herbaceous/tree and shrub), 120 (shrubland), 140 (lichen and moss), 150 (sparse vegetation), 180 (shrub/herbaceous, flooded)
Grassland	130 (grassland)
Urban	190 (urban areas)

Supplementary Table 2: **Land cover dependent spatial and temporal thresholds for merging new fire clusters with existing active fires.** Smoldering time refers to the temporal threshold, and numbers in brackets represent thresholds for the 'sleeper' procedure, where applicable.

**Land cover**	**Other**	**Cropland**	**Forest (>15% tree cover)**		**Shrubland/ mosaic/open**		**Grassland**		**Urban areas**
Peat presence			Peat	No peat	Peat	No peat	Peat	No peat	
Smoldering time (days)	4	5	10 (30)	10 (30)	10 (30)	10	10 (30)	3	5
Distance threshold (km)	1	1	2.5	2.5	2.5	2.5	1	1	1

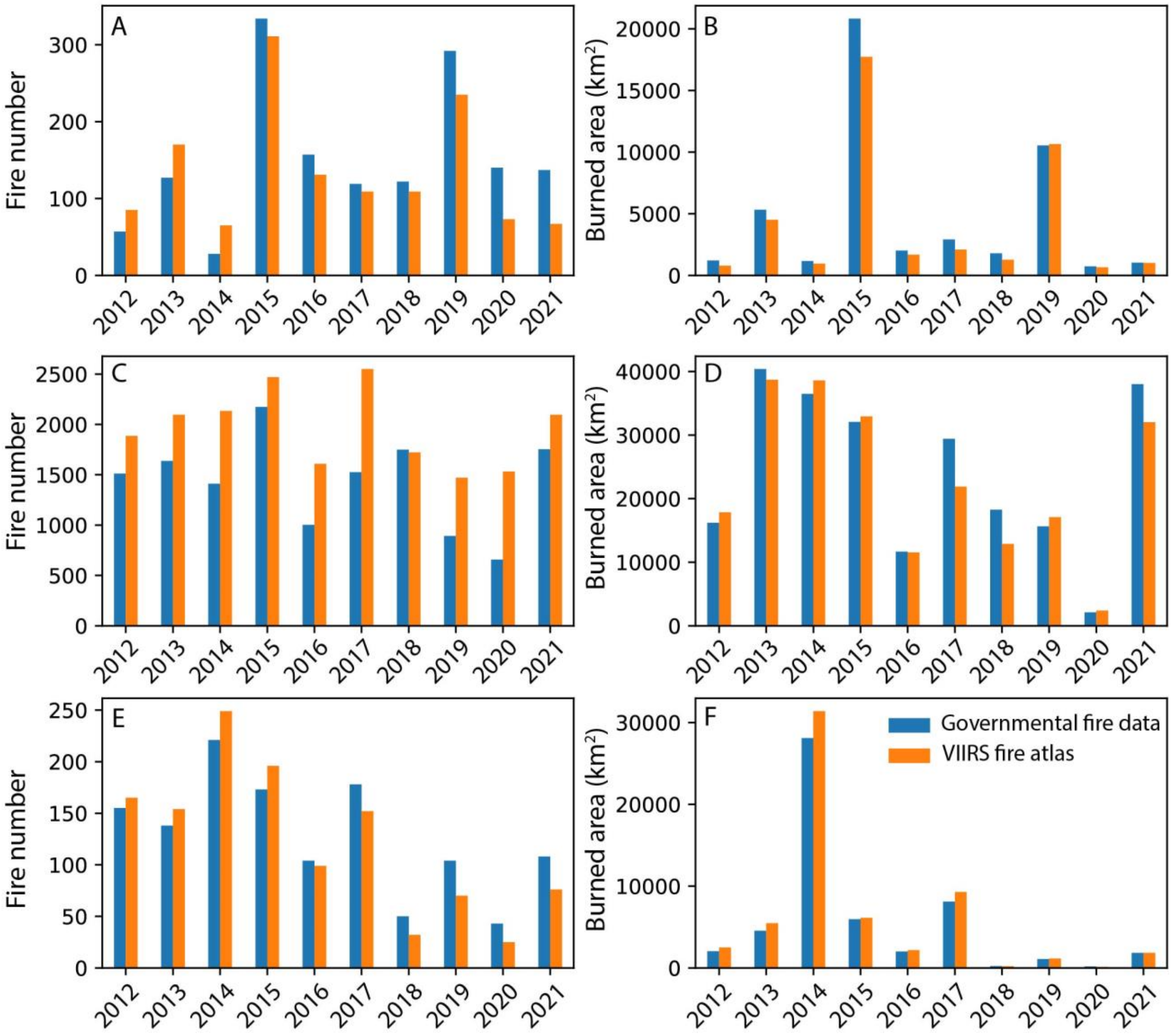

Supplementary Figure 1: **Comparison of the number of fires (A,C,E) and the annual burned area (B,D,F) of reference fire perimeters (blue) and perimeters derived from the Visible Infrared Imaging Radiometer Suite (VIIRS, orange)**. A-B: Alaska, C-D: Arctic-boreal forest and tundra regions of Canada, E-F: the Northwest Territories. Number of fires refers to the number of unique final fire perimeters, not to the number of fire starts, as this data was not available from NBAC.

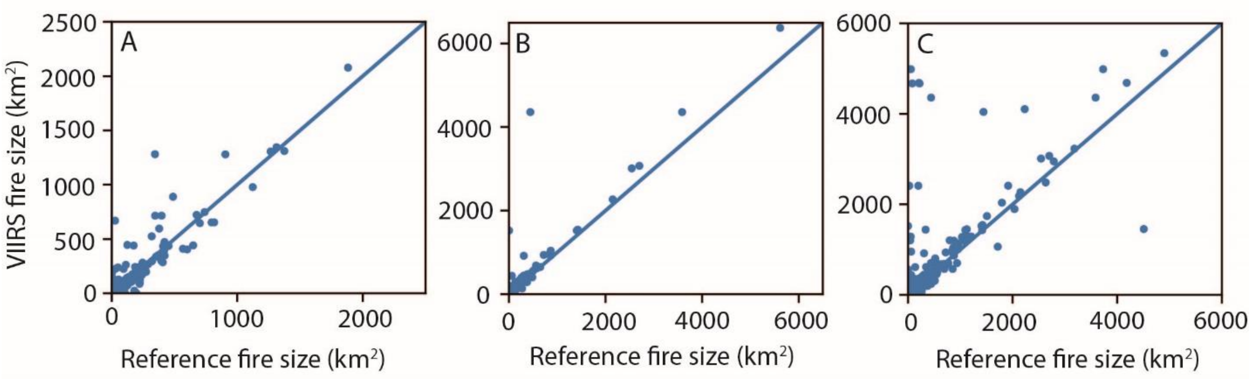

D

	Omission (%)	Commission (%)	Dif_{Mean} (km^2)	Dif_{Median} (km^2)	Dif_{Median} (%)
**Alaska**	22.4	10.7	0.96	0.45	8.1
**Canada**	28.6	24.0	13.2	0.24	10.8
**NWT, Canada**	10.4	19.5	13.9	0.54	13.3

Supplementary Figure 2: **Comparison of the size of single fires derived from Visible Infrared Imaging Radiometer Suite fire locations (VIIRS) and reference fire databases.** (A) Alaska, (B) the Northwest Territories (NWT), and (C) the Arctic-boreal forest and tundra regions of Canada. (D) Omission and commission errors of fire perimeters generated from the Visible Infrared Imaging Radiometer Suite compared to official fire databases, as well as the mean and median differences per fire.

Supplementary Table 3: **Confusion matrices comparing annual burned area derived from Visible Infrared Imaging Radiometer Suite fire locations (VIIRS) and reference fire databases**. (A) Alaska, (B) Arctic-boreal forest and tundra regions of Canada, (C) Northwest Territories.

A		***Reference (Alaska Wildland Fire Maps)***			
Alaska		*Burned (km^2)*	*Unburned (km^2)*	***Total (km^2)***	***Commission (%)***
VIIRS	*Burned (km^2)*	*36900.6*	*4419.9*	*41320.5*	*10.7*
	Unburned (km^2)	*10651.7*			
	Total (km^2)	*47552.3*			
	Omission (%)	*22.4*			

B		***Reference (National Burned Area Composite)***			
Canada		*Burned (km^2)*	*Unburned (km^2)*	***Total (km^2)***	***Commission (%)***
VIIRS	*Burned (km^2)*	*171678.3*	*54155.1*	*225833.4*	*24.0*
	Unburned (km^2)	*68755.2*			
	Total (km^2)	*240433.5*			
	Omission (%)	*28.6*			

C		***Reference (National Burned Area Composite)***			
NWT		*Burned (km^2)*	*Unburned (km^2)*	***Total (km^2)***	***Commission (%)***
VIIRS	*Burned (km^2)*	*48498.0*	*11751.5*	*60249.5*	*19.5*
	Unburned (km^2)	*5618.1*			
	Total (km^2)	*54116.1*			
	Omission (%)	*10.4*			

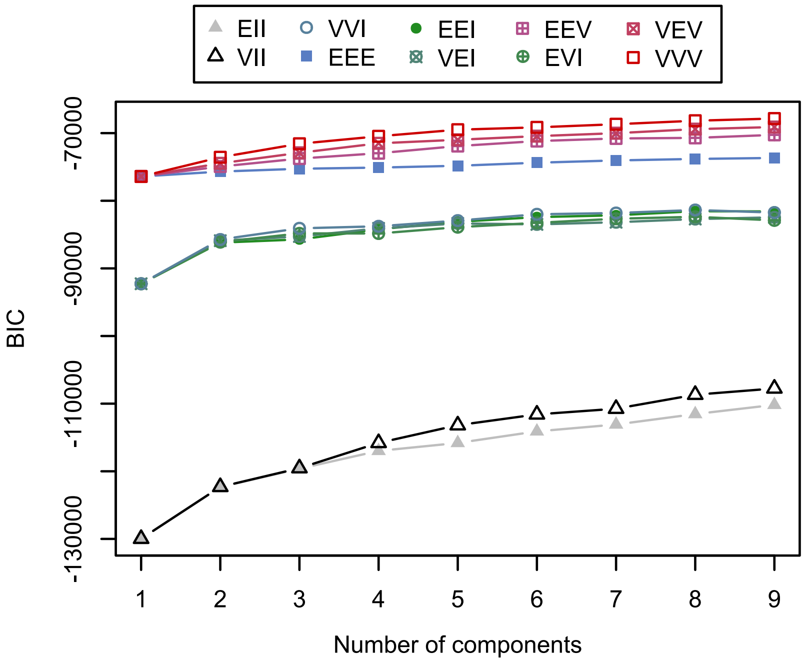

Supplementary Figure 3: **Bayesian Information Criterion (BIC) plot for models fitted with different cluster shapes and cluster numbers.** Cluster shapes are represented by different colors and shapes. Cluster shapes are defined by their volume, shape and orientation, all represented by one letter in the cluster shape name. E thereby stands for equal (all clusters can only have the same volume/shape/orientation), V for variable (values are allowed to vary per cluster), and I for invariant (in this case the shape can only represent a sphere and the orientation is along the coordinate axes).

Supplementary Table 4: **Statistics of uncertainties for the cluster affiliation of each pyroregion.** Summary statistics are based on the uncertainty assigned to each pixel by the model. N: total number of pixels, min: minimum cluster uncertainty, max: maximum cluster uncertainty, mean: average cluster uncertainty, perc90: 90th percentile of cluster uncertainty, perc95: 95th percentile of cluster uncertainties.

	**Rare Small Early**	**Rare Small Cool**	**Common Small Early**	**Common Large Cool**	**Common Large Early**	**Common Large Intense**	**Rare Small Intense**
**N**	328	456	185	394	320	568	297
**min**	0.00	0.00	0.00	0.00	0.00	0.00	0.00
**max**	0.50	0.64	0.46	0.58	0.63	0.63	0.58
**mean**	0.04	0.12	0.03	0.08	0.13	0.08	0.07
**perc90**	0.15	0.38	0.08	0.30	0.36	0.28	0.25
**perc95**	0.25	0.46	0.18	0.39	0.45	0.38	0.37

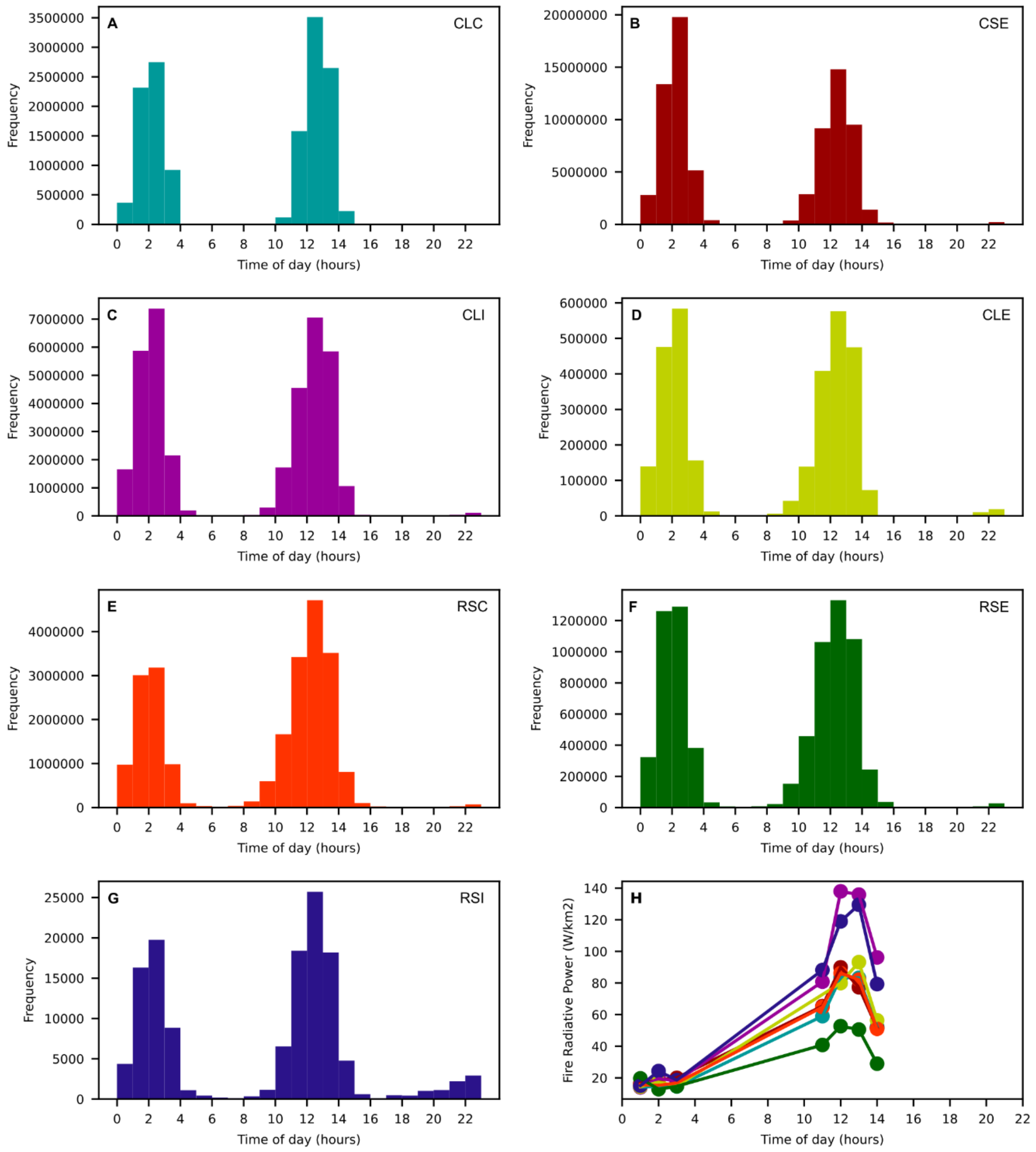

Supplementary Figure 4: **Influence of overpass time on area-normalised fire radiative power for Visible Infrared Imaging Radiometer Suite active fire locations used for the characterisation of pyroregions.** The time of day represents local solar time. A-G: Histograms of overpass times for the seven different pyroregions. A, common-large-cool, B, common-small-early, C, common-large-intense, D, common-large-early, E, rare-small-cool, F, rare-small-early, G, rare-small-intense. H: Hourly average of fire radiative power in each pyroregion. In H, we only show FRP for hourly intervals with more than 5% of the total observations.
